# Chaihu-Shugan-San for patients with nonalcoholic fatty liver disease: A systematic review and meta-analysis

**DOI:** 10.1097/MD.0000000000042303

**Published:** 2025-05-02

**Authors:** Xiangke Qu, Jianrong Sun, Yue Shen, Jia Dong, Xiaofa Li, Yanchun Ma, Jinhui Sun

**Affiliations:** a Dongzhimen Hospital, Beijing University of Chinese Medicine, Beijing, China; b Graduate School, Beijing University of Chinese Medicine, Beijing, China; c Shenzhen Traditional Chinese Medicine Hospital, Shenzhen, China; d Academic Research Department, Heilongjiang University of Chinese Medicine, Harbin, Heilongjiang Province, China.

**Keywords:** Chaihu-Shugan-San, meta-analysis, nonalcoholic fatty liver disease, systematic review

## Abstract

**Background:**

Nonalcoholic fatty liver disease (NAFLD) is a chronic liver disease characterized by intrahepatic accumulation and is closely associated with metabolic problems. Some studies have indicated that Chaihu-Shugan-San (CSS) may have a positive effect on NAFLD, but robust evidence-based research to substantiate the application of CSS is scarce. A meta-analysis was conducted to assess the clinical efficacy and safety of CSS in the treatment for NAFLD.

**Methods:**

The literature reporting CSS in NAFLD was searched from inception to October 2023 in in 7 Chinese or English databases. Studies were screened and incorporated based on predefined criteria. Data were extracted and quality was assessed independently by 2 researchers according to the Cochrane risk of bias tools. The changes in outcomes were analyzed using the mean difference (MD) and 95% confidence intervals (CIs) with a random- or fixed-effects model to examine the effect of CSS. RevMan5.4 software was used to perform meta-analyses, and the meta package of R 4.0.0 software was used for publication bias analysis.

**Results:**

A total 17 studies involving 1576 participants were screened for meta-analysis. There was high heterogeneity among studies for all continuous outcomes. Compared with common treatments, CSS could decrease aspartate-aminotransferase (MD = −12.02, 95% CI [−15.97, −8.07]), alanine-aminotransferase (MD = −10.89, 95% CI [−16.35, −5.43]), triglyceride and total cholesterol levels. In addition, CSS may increase the high-density lipoprotein cholesterol levels. And, CSS was associated with a lower incidence of adverse events (RR = 0.79, 95% CI [0.33, 1.91]).

**Conclusion:**

Current evidence shows that single or combined use of CSS is effective for NAFLD liver enzymes and blood lipids. Nevertheless, it is challenging to reach a conclusive determination owing to significant heterogeneity and ambiguous risk of bias in some trials. Therefore, more high-quality evidence is required for the clinical implementation of CSS.

## 1. Introduction

Nonalcoholic fatty liver disease (NAFLD) is a pathological condition characterized by excessive lipid accumulation in liver cells, constituting at least 5% of the liver weight, in the absence of a history of alcohol consumption and other definitive liver damage factors, such as viral infections, drug-induced liver injury, and autoimmune liver diseases.^[1,2]^ NAFLD is closely associated with various metabolic disorders, including atherosclerotic heart disease, metabolic syndrome, and type 2 diabetes mellitus (T2DM).^[3]^ Approximately 50% of individuals with T2DM are affected by NAFLD.^[4]^ The global prevalence of NAFLD ranges from 25% to 45%, with an increasing incidence in younger populations.^[5]^ Notably, the prevalence of NAFLD in China has dramatically increased from 18% to nearly 30% over the past decade, more than double that of developed countries.^[6]^ NAFLD can be divided into simple fatty liver, nonalcoholic steatohepatitis (NASH), fibrosis and cirrhosis. Simple fatty liver without significant clinical symptoms is the best stage of intervention. Fibrosis can be blocked or reversed, which is the main pathological stage affecting the prognosis of NAFLD.^[7]^ However, once the basic fatty liver progresses to liver cirrhosis, it not only increases the risk of liver cancer but is also irreversible for life, and patients might eventually require liver transplantation.^[8,9]^ Therefore, effective prevention of NAFLD occurrence and progression of NAFLD is an important public health concern. Currently, the latest guidelines recommend treatments that include lifestyle interventions, pharmacologic symptomatic treatments, and surgical treatments for end-stage cases.^[10]^ Lifestyle therapies, including exercise and dietary modifications, are recommended for NAFLD patients. However, it is hard to adhere to long-term lifestyle interventions.^[11]^ Commonly used symptomatic drug treatments include vitamin E, liver-preserving drugs (silymarin, PPCs, reduced glutathione, etc), and lipid-lowering drugs. However, there are unable to completely interrupt the progression of NAFLD.^[12]^ The Farnesoid X Receptor agonist Obeticholic Acid (OCA) has been approved by the U.S. Food and Drug Administration (FDA) for the treatment of NASH.^[13]^ Additionally, microbial therapy, including synbiotics, probiotics, and prebiotics, shows great potential for improving ALT and AST levels in adult patients with NAFLD.^[14,15]^ Surgical treatment has problems associated with postoperative infection, donor inadequacy, and postoperative recurrence. Therefore, it is of great practical significance to study how to effectively treat NAFLD.

Traditional Chinese medicine (TCM) has been used for centuries to treat patients with chronic liver diseases.^[16]^ Chaihu-Shugan-San (CSS), which was originally documented in the ancient masterpiece “*Jing-Yue book”* written by Jiebin Zhang of the Ming Dynasty, was used to improve liver depression symptoms. CSS consists of 7 herbs: Bupleuri Radix (Chaihu, *Bupleurum falcatum* L.), Paeoniae Radix Alba (Baishao, *Paeonia lactiflora Pall.*), Chuanxiong Rhizoma (Chuanxiong, *Ligusticum chuanxiong Hort*), Aurantii Fructus (Zhiqiao, *Citrus aurantium* L.), Citri Reticulatae Pericarpium (Chenpi, *Citrus reticulata Blanco*), Glycyrrhizae Radix et Rhizoma Praeparata Cum Melle (Zhigancao, *Glycyrrhiza uralensis Fisch*.), and Cyperi Rhizoma (Xiangfu, *Cyperus rotundus* L.). CSS has the effect of relaxing the liver and regulating qi, invigorating blood circulation and relieving pain. Zhang et al found that CSS reduced lipid levels and improved symptoms in patients with NAFLD.^[17]^ Some studies have indicated that CSS may significantly decrease fat accumulation in NAFLD rat livers by modulating biological processes, such as fatty acid production, insulin resistance, inflammatory response, and gut microbiology.^[18–21]^ However, the clinical effectiveness of CSS in the treatment of NAFLD is uncertain owing to a variety of problems, such as limited sample numbers, uneven research protocol design, various assessment measures, and low methodological quality. As more clinical research is carried out, it is necessary to further aggregate and update the evidence for CSS in NAFLD treatment. Therefore, we conducted a meta-analysis to assess the clinical benefits of monotherapy or combination therapy with CSS for the treatment of NAFLD, compared to conventional hepatoprotective or lipid-lowering drugs, by evaluating liver enzyme and lipid levels.

## 2. Materials and methods

The Preferred Reporting Items for Systematic reviews and Meta-Analysis (PRISMA) statement criteria were followed in this meta-analysis. The registration number for this research was CRD42024528169 in the International Prospective Register of Systematic Reviews (PROSPERO). Published clinical research was the source of all data included in this analysis.

### 2.1. Search strategy

We conducted a thorough search of Chinese databases (CNKI, the VIP database, the Wanfang Database, and SinoMed) and English databases (EMBASE, Pubmed, Cochrane Library, Clinical trials, and Web of Science) from the time of their creation until October 31, 2023. Additionally, the Cochrane Library and Clinical Trials.gov databases were searched. The searched database connections are shown in Table S1 (Supplemental Digital Content, https://links.lww.com/MD/O816, which illustrates the link of databases in this meta-analysis). Table 1 provides the Embase search approach that will also be used in other electronic databases.

**Table 1:** Search strategy used in Embase.

No.	Search items
#1	“fatty liver”/exp
#2	liver: ab, ti AND (fatty: ab, ti OR steatosis:ab,ti OR steatoses:ab,ti)
#3	NAFLD:ab,ti
#4	#1 OR #2 OR #3
#5	“chaihu shugan”:ab,ti
#6	“chaihu shugan san”/exp
#7	#5 OR #6
#8	#4 OR #7

### 2.2. Inclusion and exclusion criteria

The inclusion criteria were as follows: adult patients (age >18 years old) diagnosed with NAFLD/NASH using noninvasive techniques, such as magnetic resonance imaging (MRI), ultrasonography (US), and clinical symptoms; data from randomized controlled trials (RCTs), excluding case reports, case series, commentaries, reviews, quasiRCTs, and nonrandomized controlled studies; and study designs that included at least one group receiving a CSS intervention.

The exclusion criteria were as follows: repeated publications of the same study, and documents that did not fit the research focus.

### 2.3. Outcomes

The safety and effectiveness of CSS in the treatment of NAFLD were evaluated by analyzing liver enzymes levels, blood lipid levels, and adverse events.

Primary outcomes were liver enzymes, including alanine aminotransferase (ALT) and aspartate aminotransferase (AST).

Secondary outcomes were blood lipids and the adverse events during the experiment. Blood lipids including total cholesterol (TC), triglycerides (TG), low-density lipoprotein cholesterol (LDL-C), and high-density lipoprotein cholesterol (HDL-C).

### 2.4. Literature screening and data extraction

Two authors independently reviewed the literature, extracted the data, and verified information from the included studies. Any disputes were resolved by a third investigator. Studies unrelated to CSS in the treatment of NAFLD were discarded after skimming the title and abstract. Potentially eligible studies were identified by reading the full text. After reviewing the full text, studies that did not satisfy the inclusion criteria were excluded. The following information was extracted from each study: title, first author, country, publication year, study design, diagnostic test features, sample sizes of the case and control groups, age of the case and control subjects, country, intervention, follow-up duration, and outcomes.

### 2.5. Risk of bias assessment

Two review authors independently assessed the quality of the literature using the Cochrane Collaboration’s tool in RevMan5.4 software.^[22]^ The scoring system consists of 7 biases, including random sequence generation, allocation concealment, blinding of participants and personnel, blinding of the results assessment, incomplete data of the results, selective reporting, and other sources of bias. Each item is classified into 3 levels: low-risk, unclear, and high-risk.

### 2.6. Statistical analysis

RevMan5.4 software was used to conduct the meta-analysis. The meta package of R 4.0.0 software was used for the publication bias analysis. Continuous data were reported as mean difference (MD) and dichotomous data as relative risk (RR), with estimates and 95% confidence interval (CI). Before the outcome indicators were synthesized, heterogeneity was tested using the chi-square and Higgins *I*^2^ tests owing to natural variations in the included studies. The fixed-effects model was used when there was low heterogeneity among the included studies (*P* > .1, *I*^2^ < 50%). A random-effects model was used when the degree of heterogeneity across the studies was considerable (*P* ≤ .1, *I*^2^≥50%). If more than 10 studies were eligible, Egger test and funnel plots were performed to detect publication bias, and *P* < .05 was used to define the presence of possible publication bias.^[23]^

Subgroup analysis was used to eliminate heterogeneity, and a sensitivity analysis was performed to explore the sources of heterogeneity. Subgroup analyses were conducted according to the duration of treatment or the western medication used in the control group for indicators including more than 9 studies. Each study was sequentially removed, and the remaining studies were reanalyzed for comparison before and after deletion to clarify the stability of the results of the combined analysis.

### 2.7. Certainty of evidence assessment

The Grading of Recommendations, Assessment, Development, and Evaluation (GRADE) approach was used to assess the quality of evidence for the pooled estimates. The GRADE rating was divided into 4 levels: high, moderate, low, or very low.

## 3. Results

### 3.1. Database search and literature selection

**Figure 1.**
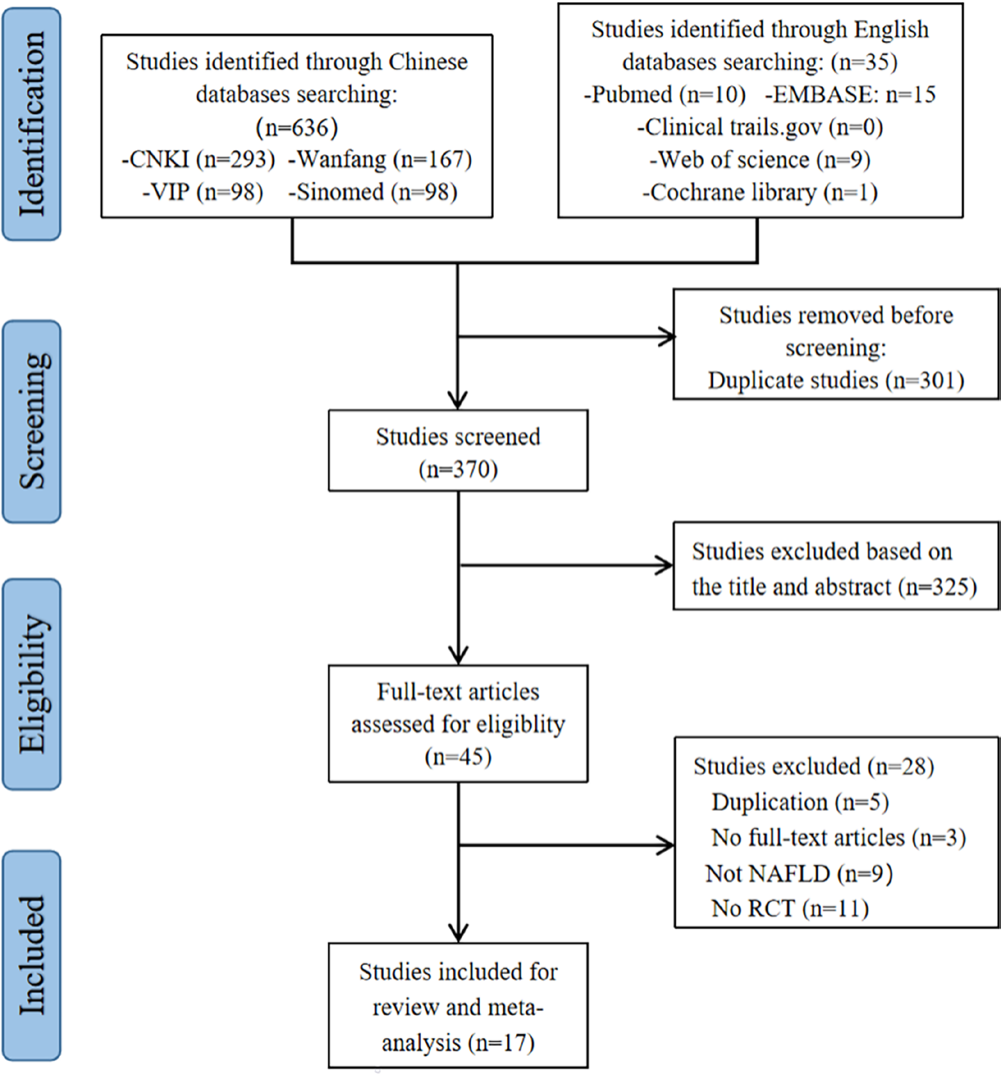
Flowchart of study selection and identification.

A comprehensive search yielded 671 articles from both Chinese and English databases. Subsequently, 301 duplicates were excluded. Forty-five studies were remained for full-text evaluation after the titles and abstracts were screened. Based on the eligibility criteria, 17 studies were included in this systematic review and meta-analysis.^[24–40]^ The literature screening process is illustrated in Figure 1.

### 3.2. Characteristics of included RCTs

This systematic review included 17 RCTs with a total of 1576 participants who were diagnosed with NAFLD and met the inclusion criteria. The sample sizes of the included studies ranged from 40 to 162. The mean age ranged between 38.49 ± 9.47 and 58.91 ± 3.05 years. The duration of treatment ranged from 30 to 180 days. All 17 studies included basic exercise and dietary therapy. Several treatment options were used in the experimental group: 7 studies used only CSS, one study used CSS and Yueju Wan, 5 studies used CSS and one basic Western medicine, and 3 studies used CSS and 2 to 4 kinds of basic Western medicine. In the control group, 9 studies used liver-protective drugs, including Dong Bao Gan Tai pills (DBGTP), Polyene Phosphatidylcholine Capsules (PPCs), and Silibinin capsules; 3 studies used anti-lipemic agents (Lovastatin, Fenofibrate, and Ezetimibe), and 3 studies used 2 to 4 kinds of basic Western medicine. One study used a hypoglycemic agent (exenatide), and the detailed characteristics are shown in Table 2.

**Table 2:** Characteristics of the included RCTs.

Study	N	Age (mean ± SD)	Sex (man/female)	Course of disease	Intervention	Time	Outcome
Pan 2009^[24]^	E: 41 C: 41	E: 44.2 ± 7.6 C: 45.3 ± 6.5	E: 29/12 C: 16/15	E: 0.5 to 15 yr C: 1 to 17 yr	E: CSS C: DBGP, 1.5 g tid	90 d	①②③④⑤⑥
Shi 2010^[25]^	E: 55 C: 55	E: 42.6 ± 0.2 C: 44.7 ± 0.3	E: 30/25 C: 31/24	E: NA C: NA	E: CSS + Lovastatin 20 mg qd C: Lovastatin, 20 mg qd	90 d	①②③④⑤⑥
Wang 2010^[26]^	E: 54 C: 54	E: 40 ± 3.7 C: 40 ± 2.6	E: 29/25 C: 27/27	E: 0.5 to 5.4 yr C: 5 mo to 5.6 yr	E: CSS C: Fenofibrate, 250 mg qd	90 d	③④⑤⑥
Teng 2011^[27]^	E: 30 C: 30	E + C: 44.6 ± 5.3	E: 20/10 C: 19/11	E + C: NA	E: CSS + PPC 456 mg tid + 228 mg tid C: PPC, 456 mg tid + 228 mg tid	90 d	①②③④⑤⑥⑦
Miao 2012^[28]^	E: 81 C: 81	E: 44.2 ± 7.6 C: 45.3 ± 6.5	E: 57/24 C: 51/30	E: 0.5 to 15 yr C: 1 to 17 yr	E: CSS + YJW C: DBGP 1.5 g tid	30 d	①②
Zhang 2013^[29]^	E: 30 C: 30	E: 44.3 ± 5.7 C: 45.2 ± 5.1	E: 18/12 C: 16/14	E: 0.5 to 10 yr C: 0.5 to 12 yr	E: CSS C: DBGP 1.5 g tid	90 d	①②③④⑤⑥
Cao 2015^[30]^	E: 36 C: 36	E: 48.7 ± 9.7 C: 51.2 ± 9.4	E: 23/13 C: 22/14	E: 4.1 ± 2.3 yr C: 3.8 ± 2.5 yr	E: CSS C: Silibinin capsule 140 mg tid	180 d	①②③④⑤⑥⑦
Fang 2016^[31]^	E: 63 C: 63	E: 58.91 ± 3.05 C: 57.82 ± 3.09	E: 46/17 C: 45/18	E: 15.62 ± 1.36 mo C:13.18 ± 1.31 mo	E: CSS + PPC 228 mg tid + ME 0.2 g bid + PB 80 mg iv qd C: PPC 228 mg tid + ME 0.2 g bid + PB 80 mg iv qd	30 d	④⑤⑥
Tan 2018^[32]^	E: 66 C: 67	E: 47.82 ± 9.36 C: 46.83 ± 10.27	E: 34/32 C: 37/30	E: 5.49 ± 1.34 yr C: 5.27 ± 1.24 yr	E: CSS + Exenatide sq 10 ug bid C: Exenatide sq 10ug bid	84 d	①②③④⑤⑥
Zhu 2018^[33]^	E:40 C: 30	E: 50.75 ± 11.63 C: 55.73 ± 10.36	E: 20/20 C: 13/17	E: 6.54 ± 2.78 yr C: 7.18 ± 3.15 yr	E: CSS C: PPC 456 mg tid + 228 mg tid	120 d	①②③④⑤⑥
Huang 2020^[34]^	E: 20 C: 20	E: 41.5 ± 5.7 C: 42.6 ± 7.1	E: 12/218 C: 13/7	E: 8.4 ± 9.7 yr C: 9.3 ± 8.9 yr	E: CSS + Atomolam 400 mg po,tid + vitamin E 100 mg po,tid C: Atomolam 400 mg po,tid + vitamin E 100 mg po,tid	168 d	①②③④⑤⑥
Luo 2020^[35]^	E: 78 C: 77	E: 47.7 ± 12.5 C: 44.3 ± 12.8	E: 53/25 C: 51/26	E: 10.15 ± 2.04 mo C: 10.41 ± 1.93 mo	E: CSS C: Silibinin capsule, 105 mg tid	84 d	①②③④
Lei 2021^[36]^	E: 40 C: 40	E；42.13 ± 5.67 C: 43.28 ± 6.67	E: 23/17 C: 25/15	E: 2.56 ± 0.55 yr C: 2.71 ± 0.61 yr	E: CSS + PPC C: PPC, 456 mg tid	90 d	①②③④⑦
Su 2021^[37]^	E: 36 C: 35	E: 49.86 ± 4.17 C: 51.60 ± 6.38	E: 20/16 C: 21/14	E: 16.72 ± 5.18 mo C: 15.73 ± 5.08 mo	E: CSS + PPC 456 mg tid + ME 0.2 g bid + Taurine Capsules 0.8 g bid + PB 80 mg iv qd C: PPC 456 mg tid + ME 0.2 g bid + Taurine Capsules 0.8 g bid + PB 80 mg iv qd	60 d	①②③④⑤⑥
Xie 2021^[38]^	E: 37 C: 35	E: 38.49 ± 9.47 C: 41.77 ± 9.12	E: 32/8 C: 28/12	E + C: NA	E: CSS C: Placebo 200 ml bid	84 d	①②③④⑤⑥⑦
Chen 2022^[39]^	E: 30 C: 30	E + C: 43.53 ± 7.71	E + C: 48/32	E + C: NA	E: CSS + Ezetimibe C: Ezetimibe 10 mg qd	168 d	①②③④⑤⑥⑦
He 2022^[40]^	E: 57 C:58	E: 47.85 ± 12.66 C: 47.56 ± 12.54	E: 31/26 C: 32/26	E + C: NA	E: CSS C: PPC 456 mg tid + 228 mg tid	120 d	①②③④⑤⑥

Abbreviations: C = control group, CSS = Chaihu-Shugan-San, DBGP = Dong Bao Gan Tai pills, E = experimental group, ME = metronidazole, PB = polymyxin B, PPC = polyene phosphatidylcholine capsule, RCTs = randomized controlled trials, YJW = Yue-Ju-Wan.

① = alanine aminotransferase, ② = aspartate aminotransferase, ③ = total cholesterol, ④ = triglycerides, ⑤ = low-density lipoprotein cholesterol, ⑥ = high-density lipoprotein cholesterol, ⑦ = adverse event.

### 3.3. Risk of bias assessment

**Figure 2.**
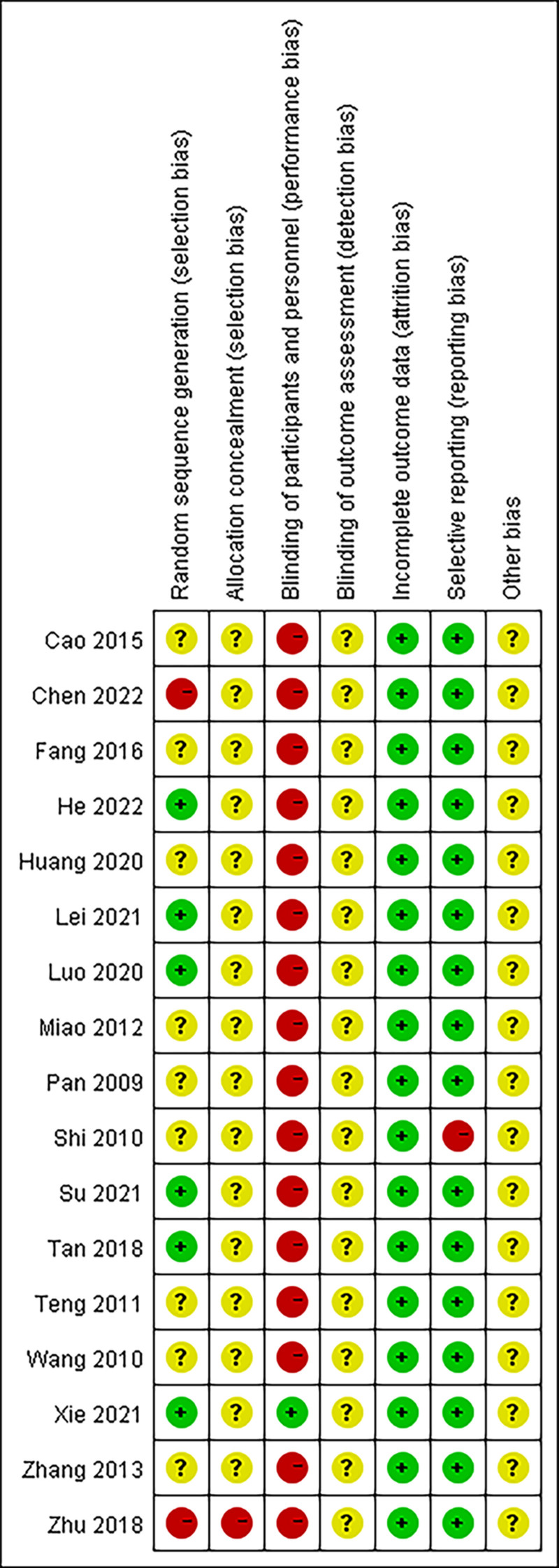
Risk of bias for the included studies. Green = low-risk; yellow = unclear; red = high-risk.

The risk of bias was assessed by the “Cocrane Collaboration’s risk of bias” assessment tool. Six studies described the randomization method used. One study reported methods for blinding participants, and blinding was not reported in the remaining studies. A summary and graph of the risk of bias are shown in Figure 2.

### 3.4. Meta-analysis and narrative review

In Xie study, all continuous outcomes were nonnormally distributed.^[35]^ Therefore, we performed descriptive analysis of the data. They found that ALT, AST, TC, and LDL-C levels were lower after CSS intervention than after placebo intervention (*P* < .05). The HDL-C level was greater than that in the placebo group (*P* < .05). However, there were no significant differences in the TG level between the 2 groups.

#### 3.4.1. Liver enzymes

Fourteen RCTs reported evaluable ALT and AST data, including 641 participants and 629 in the in the experimental and control groups, respectively.

**Figure 3.**
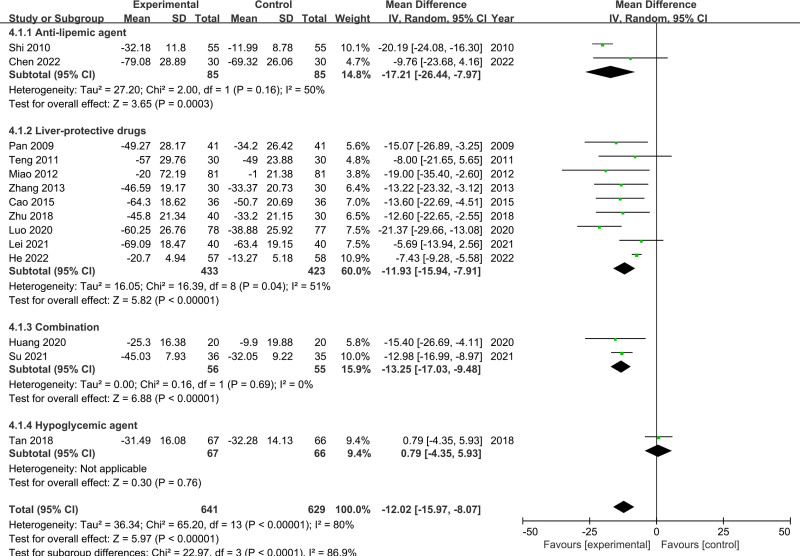
Meta-analysis results for ALT levels. ALT = alanine aminotransferase.

Because of significant study heterogeneity (*P* < .00001, *I*^2^ = 80%), a random-effects model was used for ALT. The results showed that the CSS patients had significantly lower ALT levels than the controls (MD = −12.02, 95% CI [−15.97, −8.07], *P* < .00001) (Fig. 3). To analyze the source of ALT heterogeneity, a subsequent subgroup analysis was conducted. The results showed that the medicine used in the control group was an important reason for heterogeneity (*P* < .0001, *I*^2^ = 86.9%). The results of the sensitivity analysis revealed that the heterogeneity decreased (*I*^2^ from 80% to 48%) after the removal of the study by Shi and Tan. However, there was no change in the conclusion of the meta-analysis when 2 studies were removed (MD = −12.02, 95% CI [−15.15, −8.89], *P* < .00001), which suggested that the findings were stable and reliable.

**Figure 4.**
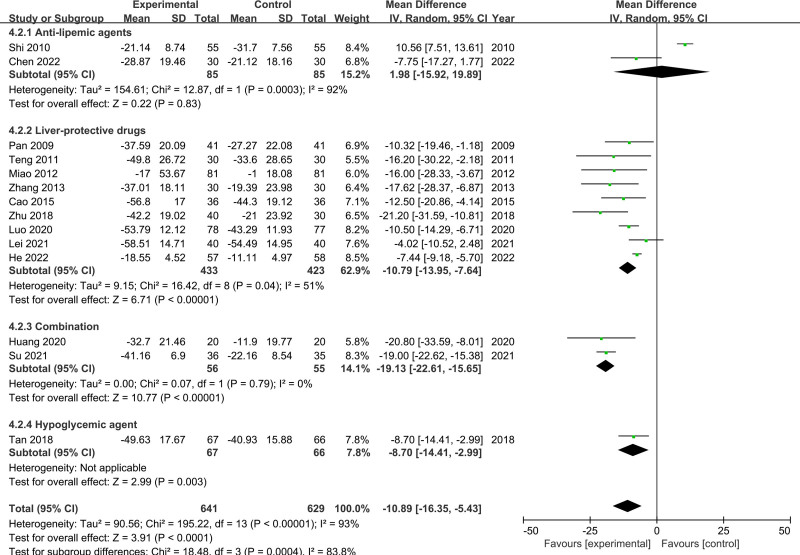
Meta-analysis results for AST levels. AST = aspartate aminotransferase.

The heterogeneity test for AST revealed considerable heterogeneity (*I*^2^ = 93%, *P* < .00001), which was analyzed using a random effects model. The results showed that the AST levels were significantly lower in the CSS group than in the control group (MD = −10.89, 95% CI [−16.35, −5.43], *P* < .00001) (Fig. 4). The results of the subgroup analysis indicated that the medicine used in the control group was an important reason for heterogeneity (*P* < .0004, *I*^2^ = 83.8%). In addition, the results of sensitivity analysis showed the heterogeneity decreased (*I*^2^ from 93% to 48%) after the removal of the study by Shi, and Su. When these studies were removed from the meta-analysis, there was no significant change in the findings (MD = −10.62, 95% CI [−13.28, −7.96], *P* < .00001).

#### 3.4.2. Blood lipids

**Figure 5.**
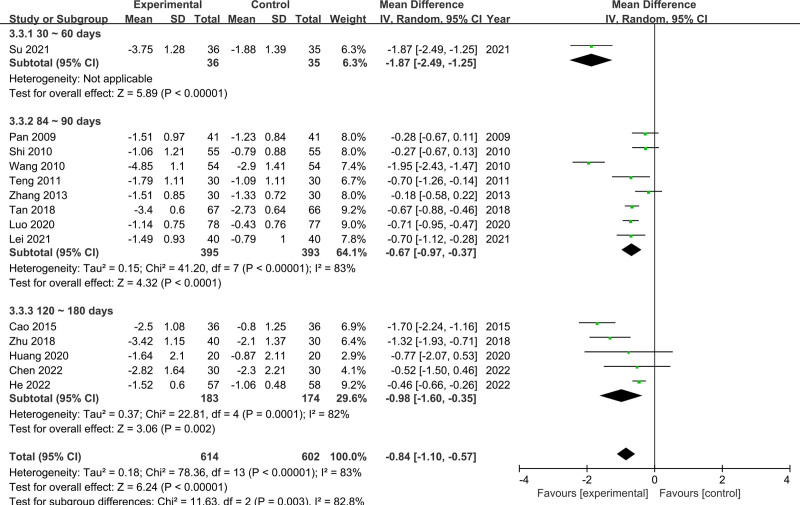
Meta-analysis results for TC levels. TC = total cholesterol.

Fourteen RCTs involving 614 participants in the experimental group and 602 in the control group reported evaluable TC data. The random-effects model was used because of the substantial heterogeneity among the studies (*P* < .00001, *I*^2^ = 83%). The results showed that the CSS group had lower TC levels than control group (MD = −0.84, 95% CI [−1.10, −0.57]) (Fig. 5). The subgroup analysis revealed that the duration of treatment was responsible for the heterogeneity (*P* = .003, *I*^2^ = 82.8%). After performing a sensitivity analysis, the results showed that the heterogeneity decreased among the remaining studies after the removal of the studies by Wang, Cao, and Lei (*I*^2^ from 83% reduced to 44%). The meta-analysis results changed after removing these studies (MD = −0.56, 95% CI [−0.71, −0.40], *P* < .00001).

**Figure 6.**
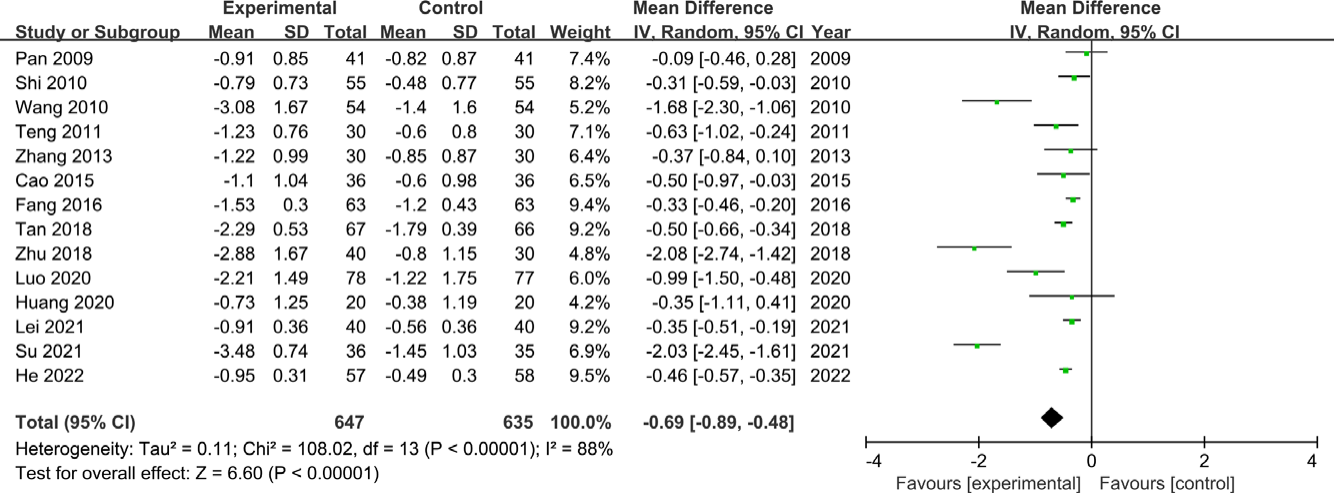
Results of the meta-analysis of TG levels. TG = triglycerides.

Fourteen studies included TG indicators, involving 647 participants in the experimental group and 635 participants in the control group. The random-effects model was used for the analysis because of considerable heterogeneity (*I*^2^ = 88%, *P* < .00001). The results indicated that CSS reduced TC levels compared to the control treatment (MD = −0.69, 95% CI [−0.89, −0.48]) (Fig. 6). The subgroup analysis did not find the reason for heterogeneity among studies based on the duration of follow-up and western medicine used in the control group. However, the results of sensitivity analysis showed that the heterogeneity decreased (*I*^2^ from 88% to 27%) after the removal of the study by Wang, Zhu, and Su. Unfortunately, the results of the meta-analysis changed after these studies were removed (MD = −0.41 95% CI [−0.50, −0.33], *P* < .00001).

**Figure 7.**
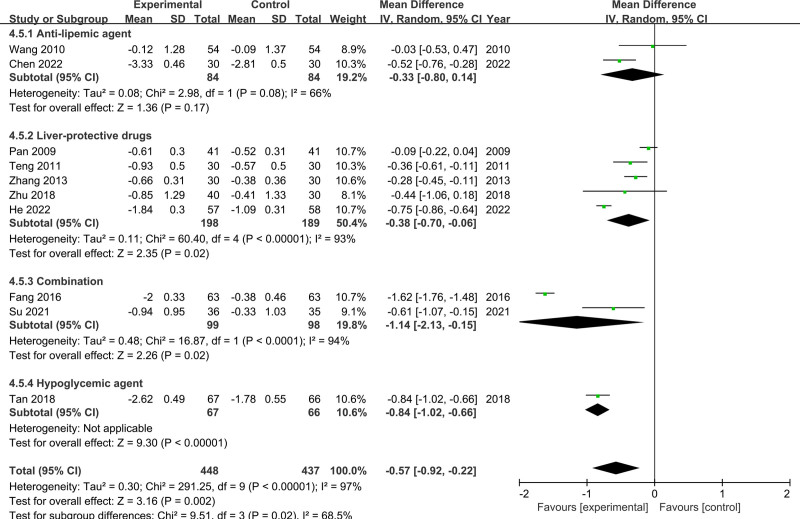
Meta-analysis results of LDL-C levels. LDL-C = low-density lipoprotein cholesterol.

Ten RCTs reported evaluable LDL-C data, including 448 participants in the experimental group and 437 participants in the control group. The random-effects model was used for analysis because of the high heterogeneity among the studies (*I*^2^ = 97%, *P* < .00001). The results showed that LDL-C level was lower in the CSS group than in the control group (MD = −0.57, 95% CI [−0.92, −0.22]) (Fig. 7). The subgroup analysis found that the medicine used in the control group was an influential factor for heterogeneity (*P* = .02, *I*^2^ = 68.5%). The sensitivity analysis indicated that the heterogeneity was decreased (*I*^2^ from 97% to 7%, *P* = .37) with the exclusion of the studies by Pang, Fang, Tan, and He. In addition, the results of the meta-analysis changed after these studies were removed (MD = −0.36, 95% CI [−0.48, −0.24], *P* < .00001).

**Figure 8.**
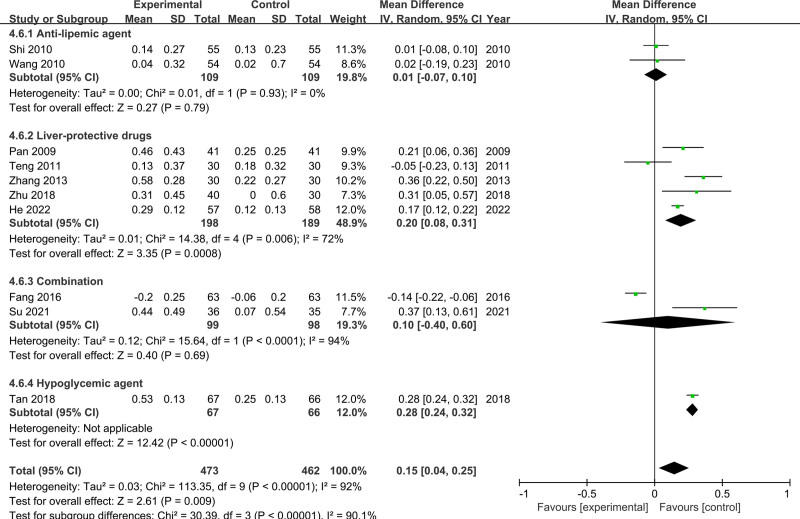
Meta-analysis results for HDL-C levels. HDL-C = high-density lipoprotein cholesterol.

Ten studies reported evaluable HDL-C data, involving 473 participants in the experimental group and 462 in the control group. The random-effects model was used for the analysis based on considerable heterogeneity (*I*^2^ = 92%, *P* < .00001). The results showed that CSS improved HDL-C levels compared to the control group (MD = 0.15, 95% CI [0.04, 0.25]) (Fig. 8). The subgroup analysis found that the medicine used in the control group was the main source of heterogeneity (*P* < .00001, *I*^2^ = 90.1%). In addition, the sensitivity analysis found the heterogeneity was decreased (*I*^2^ from 92% to 34%) after the removal of the study by Shi, Teng, Zhang, Fan, and Tan. The results of the meta-analysis did not change significantly after these studies were removed (MD = 0.19, 95% CI [0.11, 0.27], *P* < .00001).

#### 3.4.3. Adverse events

**Figure 9.**
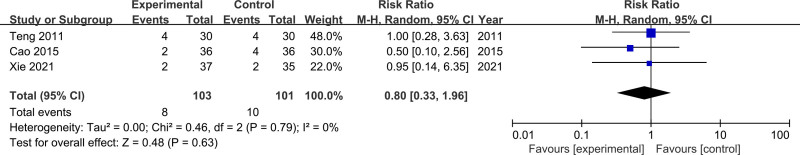
Meta-analysis results of adverse events.

Among the 17 studies, only 4 reported the occurrence or absence of adverse reactions, and the remaining studies did not report adverse reactions. One study reported no serious adverse events in either the experimental or control groups. Three studies were included in this meta-analysis (Fig. 9). Considering the limited number of studies incorporated in the analysis, a random-effects model was employed to account for potential variability across studies. The RR of experiencing any adverse event was lower with CSS (RR = 0.80, 95% CI [0.33, 1.96]).

### 3.5. Publication bias

All continuous indicators were included in more than 10 studies. Egger test and funnel plots were performed to analyze publication bias. The results of the Egger test showed that ALT (*P* = .2797), AST (*P* = .3085), TC (*P* = .1561), LDL-C (*P* = .4579), and HDL-C (*P* = .5140) levels had no significant publication bias. However, TG (*P* = .0558) may present some potential publication bias. The funnel plots are shown in Figures S1 to S6 (Supplemental Digital Content, https://links.lww.com/MD/O817, which displaying the funnel plots for liver enzyme and lipid profile indicators).

### 3.6. Certainty of evidence assessment

The GRADE approach was employed to ascertain the quality of evidence for each outcome measure. Our evaluation revealed that the quality of evidence for the outcome measures, including ALT, AST, TC, LDL-C, HDL-C, and AE, was consistently rated as low (Table 3). Furthermore, the evidence quality for TG was deemed to be very low, indicating a greater degree of uncertainty and higher potential for bias or imprecision in the findings.

**Table 3:** Summary of GRADE evidence quality assessment.

Outcome	Number of participants (studies)	Effect estimate (95% CI)	Risk of bias	Inconsistency	Indirectness	Imprecision	Publication bias	GRADE
ALT	1270 (14)	MD −12.02 (−15.97, −8.07)	Suspected	Suspected	No serious	No serious	No serious	⊕⊕⊖⊖Low
AST	1270 (14)	MD −10.89 (−16.35, −5.43)	Suspected	Suspected	No serious	No serious	No serious	⊕⊕⊖⊖Low
TC	1216 (14)	MD −0.84 (−1.10, −0.57)	Suspected	Suspected	No serious	No serious	No serious	⊕⊕⊖⊖Low
TG	1282 (14)	MD −0.69 (−0.89, −0.48)	Suspected	Suspected	No serious	No serious	Suspected	⊕⊖⊖⊖Very low
LDL-C	885 (10)	MD −0.57 (−0.92, −0.22)	Suspected	Suspected	No serious	No serious	No serious	⊕⊕⊖⊖Low
HDL-C	935 (10)	MD 0.15 (0.04, 0.25)	Suspected	Suspected	No serious	No serious	No serious	⊕⊕⊖⊖Low
AE	204 (3)	RR 0.80 (0.33, 1.96)	Suspected	No serious	No serious	Suspected	No serious	⊕⊕⊖⊖Low

Abbreviations: AE = adverse event, ALT = alanine aminotransferase, AST = aspartate aminotransferase, CI = confidence intervals, HDL-C = high-density lipoprotein cholesterol, LDL-C = low-density lipoprotein cholesterol, MD = mean difference, RR = relative risk, TC = total cholesterol, TG = triglycerides.

## 4. Discussion

TCM, deeply rooted in the rich tapestry of traditional Chinese practices, offers a compelling alternative approach to clinical treatment through its foundational “syndrome differentiation and treatment” principles. This approach not only enhances liver lipid metabolism but also mitigates inflammation and addresses fibrosis, showcasing its potential as a valuable therapeutic option.^[41,42]^ CSS is a traditional Chinese herbal medicine formula that has been used to treat a variety of chronic diseases, including depression, chronic gastritis, chronic liver disease, and functional dyspepsia.^[43–45]^ In 2014, Zhang et al conducted 10 RCTs with 802 subjects, and the results of their meta-analysis showed the better beneficial effects of CSS than the control group.^[17]^ However, the recently published studies still need further evaluation to provide more effective information for clinical use. In the present study, we systematically assessed the efficacy and safety of CSS in patients with NAFLD, including the latest studies.

Seventeen RCTs were included in this meta-analysis and systematic review. The results showed that CSS was able to significantly decrease the levels of ALT, AST, TC, TG and LDL-C compared with those in the control, which is a similar conclusion to that of a previous study. In addition, this study revealed that CSS may increase the HDL-C level, which plays a protective role in blood pressure and vessels. Moreover, CSS did not increase the incidence of adverse events. These results indicate that CSS may be effective in the treatment of mild NAFLD and is highly safe.

A potential causal link exists between depression and NAFLD.^[46]^ Studies have reported that the prevalence of depression among individuals with NAFLD ranges from 18.21% to 26.1%, highlighting the significant comorbidity between these 2 conditions.^[47,48]^ CSS has demonstrated efficacy in alleviating both NAFLD and depressive symptoms, indicating its potential as a therapeutic candidate for patients with NAFLD and concurrent depression. This could pave the way for novel alternatives to traditional psychotropic medications, potentially minimizing their use. In addition, network pharmacology and molecular docking suggest the potential therapeutic effects of CSS in liver fibrosis and hepatocellular carcinoma.^[49,50]^ Further evidence is required to clarify these findings.

All indicators had high heterogeneity in this study, which may have resulted from different baseline clinical characteristics and intervention designs among the included studies. In the subsequent sensitivity analysis, after 2 to 5 studies were rejected, heterogeneity was removed, and some results of the meta-analysis were changed. In the subgroup analysis, the single or combined use of CSS improved ALT and AST levels more effectively than liver-protective drugs. A reasonable treatment time may be the key to the efficacy of CSS.

The GRADE assessment results showed that the quality of the present meta-analysis was unsatisfactory, hence it is important to consider the study’s findings and conclusions cautiously. Firstly, 6 of the included studies reported the use of randomization methods, and only one article reported blinding of participants and personnel methods. Thus, it might create a potential bias in the selection of cases and distribution of interventions. Secondly, the lack of uniform standards for the relevant components, quality control, dosage, and usage of the CSS formula which has an impact on the comparability among the included studies. Thirdly, the included papers were all published in Chinese and did not include any potentially unfavorable literature that was not publicly available, which may have some risk of publication bias. Therefore, more high-quality, large-sample trials with placebo-controlled groups in different countries are needed to validate the effect of CSS on NAFLD.

## 5. Conclusion

In conclusion, CSS is both effective and safe for the treatment of NAFLD when used alone or in combination with Western medications. However, the high heterogeneity and poor quality of the studies included in this meta-analysis could have had a significant impact on the findings. More standardized clinical trials are required to verify this speculation. If the favorable effect of CSS is verified in future high-level clinical trials, it could be used as a complementary therapy for NAFLD treatment.

## Acknowledgments

The authors would like to express their gratitude to the Beijing University of Chinese Medicine Library for providing access to valuable literature search resources.

## Author contributions

**Conceptualization:** Jinhui Sun.

**Data curation:** Yue Shen.

**Methodology:** Xiangke Qu, Jia Dong.

**Project administration:** Jinhui Sun.

**Software:** Jianrong Sun, Jia Dong.

**Supervision:** Jinhui Sun.

**Validation:** Yue Shen.

**Visualization:** Jianrong Sun, Yue Shen.

**Writing – original draft:** Xiangke Qu, Jianrong Sun.

**Writing – review & editing:** Xiangke Qu, Jia Dong, Xiaofa Li, Yanchun Ma.

## Supplementary Material



## References

[1] Powell EE Wong VW Rinella M. Non-alcoholic fatty liver disease. Lancet. 2021;397:2212–24. 33894145 10.1016/S0140-6736(20)32511-3

[2] Younossi ZM. Non-alcoholic fatty liver disease – a global public health perspective. J Hepatol. 2019;70:531–44. 30414863 10.1016/j.jhep.2018.10.033

[3] Golabi P Fukui N Paik J Sayiner M Mishra A Younossi ZM. Mortality risk detected by atherosclerotic cardiovascular disease score in patients with nonalcoholic fatty liver disease. Hepatol Commun. 2019;3:1050–60. 31388626 10.1002/hep4.1387 PMC6671783

[4] Kim KS Hong S Han K Park C-Y. Association of non-alcoholic fatty liver disease with cardiovascular disease and all cause death in patients with type 2 diabetes mellitus: nationwide population based study. BMJ. 2024;384:e076388. 38350680 10.1136/bmj-2023-076388 PMC10862140

[5] Younossi Z Anstee QM Marietti M. Global burden of NAFLD and NASH: trends, predictions, risk factors and prevention. Nat Rev Gastroenterol Hepatol. 2018;15:11–20. 28930295 10.1038/nrgastro.2017.109

[6] Zhou J Zhou F Wang W. Epidemiological features of NAFLD From 1999 to 2018 in China. Hepatology. 2020;71:1851–64. 32012320 10.1002/hep.31150

[7] Cotter TG Rinella M. Nonalcoholic fatty liver disease 2020: the state of the disease. Gastroenterology. 2020;158:1851–64. 32061595 10.1053/j.gastro.2020.01.052

[8] Juanola O Martínez-López S Francés R Gómez-Hurtado I. Non-alcoholic fatty liver disease: metabolic, genetic, epigenetic and environmental risk factors. Int J Environ Res Public Health. 2021;18:5227. 34069012 10.3390/ijerph18105227 PMC8155932

[9] Ginès P Krag A Abraldes JG Solà E Fabrellas N Kamath PS. Liver cirrhosis. Lancet. 2021;398:1359–76. 34543610 10.1016/S0140-6736(21)01374-X

[10] European Association for the Study of the Liver (EASL); European Association for the Study of Diabetes (EASD); European Association for the Study of Obesity (EASO); European Association for the Study of the Liver (EASL). EASL-EASD-EASO Clinical Practice Guidelines on the management of metabolic dysfunction-associated steatotic liver disease (MASLD). J Hepatol. 2024;81:492–542. 38851997 10.1016/j.jhep.2024.04.031

[11] Xanthakos SA Lavine JE Yates KP; NASH Clinical Research Network. Progression of fatty liver disease in children receiving standard of care lifestyle advice. Gastroenterology. 2020;159:1731–51.e10. 32712103 10.1053/j.gastro.2020.07.034 PMC7680281

[12] Oh H Jun DW Saeed WK Nguyen MH. Non-alcoholic fatty liver diseases: update on the challenge of diagnosis and treatment. Clin Mol Hepatol. 2016;22:327–35. 27729634 10.3350/cmh.2016.0049 PMC5066376

[13] Neuschwander-Tetri BA Loomba R Sanyal AJ; NASH Clinical Research Network. Farnesoid X nuclear receptor ligand obeticholic acid for non-cirrhotic, non-alcoholic steatohepatitis (FLINT): a multicentre, randomised, placebo-controlled trial. Lancet. 2015;385:956–65. 25468160 10.1016/S0140-6736(14)61933-4 PMC4447192

[14] Musazadeh V Assadian K Rajabi F. The effect of synbiotics on liver enzymes, obesity indices, blood pressure, lipid profile, and inflammation in patients with non-alcoholic fatty liver: a systematic review and meta-analysis of randomized controlled trials. Pharmacol Res. 2024;208:107398. 39241935 10.1016/j.phrs.2024.107398

[15] Kanchanasurakit S Kositamongkol C Lanoi K. Effects of synbiotics, probiotics, and prebiotics on liver enzymes of patients with non-alcoholic fatty liver disease: a systematic review and network meta-analysis. Front Nutr. 2022;9:880014. 35669067 10.3389/fnut.2022.880014 PMC9167056

[16] Ji L Li Q He Y. Therapeutic potential of traditional Chinese medicine for the treatment of NAFLD: a promising drug Potentilla discolor Bunge. Acta Pharm Sin B. 2022;12:3529–47. 36176915 10.1016/j.apsb.2022.05.001 PMC9513494

[17] Zhang LD Sun XH Wei W. Systematic evaluation and meta analysis on nonalcoholic fatty liver disease treated with Chaihu Shugan San. World J Integr Tradit West Med. 2014;9:1004–7.

[18] Zheng C Nie H Pan M. Chaihu Shugan powder influences nonalcoholic fatty liver disease in rats in remodeling microRNAome and decreasing fatty acid synthesis. J Ethnopharmacol. 2024;318(Pt A):116967. 37506783 10.1016/j.jep.2023.116967

[19] Lei S Zhao S Huang X. Chaihu Shugan powder alleviates liver inflammation and hepatic steatosis in NAFLD mice: a network pharmacology study and in vivo experimental validation. Front Pharmacol. 2022;13:967623. 36172180 10.3389/fphar.2022.967623 PMC9512055

[20] Jiang WN Li D Jiang T. Protective effects of chaihu shugan san () on nonalcoholic fatty liver disease in rats with insulin resistance. Chin J Integr Med. 2018;24:125–32. 27164963 10.1007/s11655-016-2252-4

[21] Liang Y Zhang Y Deng Y. Chaihu-Shugan-San Decoction modulates intestinal microbe dysbiosis and alleviates chronic metabolic inflammation in NAFLD rats via the NLRP3 inflammasome pathway. Evid Based Complement Alternat Med. 2018;2018:9390786. 30105078 10.1155/2018/9390786 PMC6076928

[22] Higgins JP Altman DG Gøtzsche PC; Cochrane Bias Methods Group. The Cochrane Collaboration’s tool for assessing risk of bias in randomised trials. BMJ. 2011;343:d5928. 22008217 10.1136/bmj.d5928 PMC3196245

[23] Egger M Davey Smith G Schneider M Minder C. Bias in meta-analysis detected by a simple, graphical test. BMJ. 1997;315:629–34. 9310563 10.1136/bmj.315.7109.629 PMC2127453

[24] Pan FM Huang JR. Clinical observation of Chaihu Shugan San in the treatment of 82 cases of non-alcoholic fatty liver. Lishizhen Med Materia Medica Res. 2009;20:2010–1.

[25] Shi QJ. Chaihu Shugan powder on treating 55 cases of fatty liver. J Pract Tradit Chin Intern Med. 2010;24:47–8.

[26] Wang XW Wang D Wang Z. Clinical investigation of Chaihu shugan san on non-alcoholic fatty liver. Hebei J Tradit Chin Med. 2010;32:1129–31.

[27] Teng XS. Efficacy of combined treatment of traditional Chinese medicine and western medicine for non-alcoholic fatty liver disease. Chin J Prim Med Pharm. 2011;18:1299–301.

[28] Miao XD. Chaiyue Decoction for 81 cases with nonalcoholic fatty liver. Shandong J Tradit Chin Med. 2012;31:640–2.

[29] Zhang L. Clinical observation on therapeutic effect of Chaihu Shugan San in Treating non-alcoholic fatty liver. Chin Med Modern Distance Educ China. 2013;11:90–1.

[30] Cao FL. Observation on therapeutic effect of Chaihu Shugan San in Treating Nonalcoholic Fatty Liver Disease. Guangming J Chin Med. 2015;30:746–9.

[31] Fang QXX. Combining traditional Chinese and western medicine for nonalcoholic fatty liver diseases with liver qi stagnation syndrome. J Pract Tradit Chin Med. 2016;32:344–5.

[32] Tan R Deng ZR Ding XH. Effect of Chaihu Shugan powder combined with Exenatide on quantitative index of total liver fat in patients with non-alcoholic fatty liver disease. Laborat Med Clin. 2018;15:1998–2001.

[33] Zhu N Zhao XJ Tan BB Qin FX. Clinical study on Chaihu Shugan Powder in treating non-alcoholic fatty liver. Shenzhen J Integr Tradit Chin West Med. 2018;28:56–7.

[34] Huang LP Chen Y. The clinical effect of Chaihu-Shugan powder on nonalcoholic fatty liver based on FibroScan Technology. J Qingyuan Polytech. 2020;13:35–8.

[35] Luo MC Xue XX Chen XF. Clinical study on the effect of Chaihu Shugan Powder for non-alcoholic steatohepatitis inflammatory factors. Tianjin J Tradit Chin Med. 2020;37:187–92.

[36] Lei YJ Wang LL Y XD. Clinical study on modified Chaihu Shugan powder combined with Polyene phosphati-dylcholine capsules for non-alcoholic steatohepatitis. J New Chin Med. 2021;53:24–8.

[37] Su W. Clinical effect of Chaihu Shuan powder for patients with non-alcoholic fatty liver. Pract Clin J Integr Tradit Chin West Med. 2021;21:8–9.

[38] Xie WN Peng HB Li Y. Liver with Liver Stagnation and Spleen Deficiency Syndrome and intestinal microflora. Chin J Exp Tradit Med Formulae. 2021;27:129–37.

[39] Chen W Li X Wang Y Wang LW. Observation on the clinical effect of Ezetimibe Combined with Chaihu Shugan Pill in the treatment of nonalcoholic fatty liver disease. Guangming J Chin Med. 2022;37:3194–6.

[40] He SY Chen CY Huang JF. Therapeutic effect and mechanism of Chaihu Shugansan on nonalcoholic fatty liver disease. J North China Univ Sci Technol. 2022;24:221–5.

[41] Zhang WY Wang MH Xie C. Potential of traditional Chinese medicine in the treatment of nonalcoholic fatty liver disease: a promising future. World J Gastroenterol. 2024;30:4597–601. 39575403 10.3748/wjg.v30.i43.4597 PMC11572638

[42] Liu Y Fan Y Liu J Liu X Li X Hu J. Application and mechanism of Chinese herb medicine in the treatment of non-alcoholic fatty liver disease. Front Pharmacol. 2024;15:1499602. 39605910 10.3389/fphar.2024.1499602 PMC11598537

[43] Zhang X Zhao Q Wang Y Mao Y Sun Y Bian X. Effectiveness and safety of Chaihu-Shugan-San for treating depression based on clinical cases: an updated systematic review and meta-analysis. Medicine (Baltim). 2024;103:e38668. 10.1097/MD.0000000000038668 PMC11466128 38941409

[44] Qin F Liu JY Yuan JH. Chaihu-Shugan-San, an oriental herbal preparation, for the treatment of chronic gastritis: a meta-analysis of randomized controlled trials. J Ethnopharmacol. 2013;146:433–9. 23376045 10.1016/j.jep.2013.01.029

[45] Wang Y Jia Y Liu X. Effect of Chaihu-Shugan-San on functional dyspepsia and gut microbiota: a randomized, double-blind, placebo-controlled trial. J Ethnopharmacol. 2024;322:117659. 38151181 10.1016/j.jep.2023.117659

[46] Zhou X Liao J Liu L. Association of depression with severe non-alcoholic fatty liver disease: evidence from the UK Biobank study and Mendelian randomization analysis. Sci Rep. 2024;14:28561. 39557910 10.1038/s41598-024-79100-z PMC11574024

[47] Xiao J Lim LKE Ng CH. Is fatty liver associated with depression? A meta-analysis and systematic review on the prevalence, risk factors, and outcomes of depression and non-alcoholic fatty liver disease. Front Med (Lausanne). 2021;8:691696. 34277666 10.3389/fmed.2021.691696 PMC8278401

[48] Shea S Lionis C Kite C. Non-alcoholic fatty liver disease and coexisting depression, anxiety and/or stress in adults: a systematic review and meta-analysis. Front Endocrinol (Lausanne). 2024;15:1357664. 38689730 10.3389/fendo.2024.1357664 PMC11058984

[49] Xie Z Xie Z Trujillo NP Yang T Yang C. Exploring mechanisms of Chaihu-Shugan-San against liver fibrosis by integrated multi-omics and network pharmacology approach. Biosci Rep. 2022;42:BSR20221030. 35791909 10.1042/BSR20221030 PMC9301292

[50] Xing JH Tan RX Huang FE Tian N. Integrated analyses for identification of a three-gene signature associated with Chaihu Shugan San formula for hepatocellular carcinoma treatment. J Cell Mol Med. 2024;28:e18211. 38613352 10.1111/jcmm.18211 PMC11015397

