# Supplementary material for: Chaihu-Shugan-San for patients with nonalcoholic fatty liver disease: A systematic review and meta-analysis
Source: Medicine (Baltimore). 2025 May 2;104(18):e42303. doi: 10.1097/MD.0000000000042303 (PMC12055143; doi:10.1097/MD.0000000000042303)
Supplement: Supplementary file 1 [file medi-104-e42303-s001.docx]

**Table S1 The link of databases in this meta-analysis**

| Databases | Link |
| --- | --- |
| CNKI | <http://www.cnki.net/> |
| WanFang | [https://www.wanfangdata.com.cn/](https://www.wanfangdata.com.cn/) |
| VIP | <http://www.cqvip.com/> |
| Sinomed | <https://www.sinomed.ac.cn/> |
| EMbase | <https://www.elsevier.com/solutions/embase> |
| Pubmed | <https://pubmed.ncbi.nlm.nih.gov/> |
| Cochrane Library | <https://www.cochranelibrary.com/> |
| Clinical Trials | <https://clinicaltrials.gov/> |
| Web of Science | <https://www.webofscience.com/> |
