# Supplementary material for: Chaihu-Shugan-San for patients with nonalcoholic fatty liver disease: A systematic review and meta-analysis
Source: Medicine (Baltimore). 2025 May 2;104(18):e42303. doi: 10.1097/MD.0000000000042303 (PMC12055143; doi:10.1097/MD.0000000000042303)
Supplement: Supplementary file 2 [file medi-104-e42303-s002.pdf]

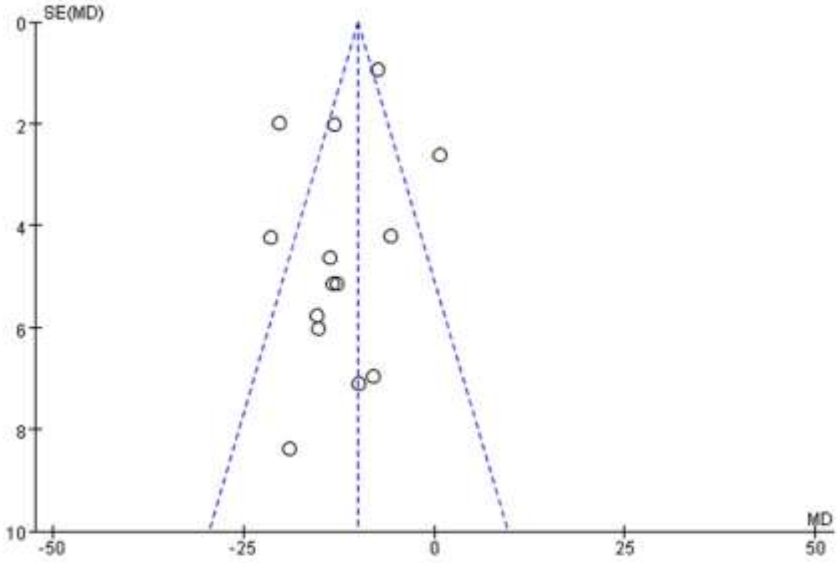

FigueS1 Funnel plot of ALT

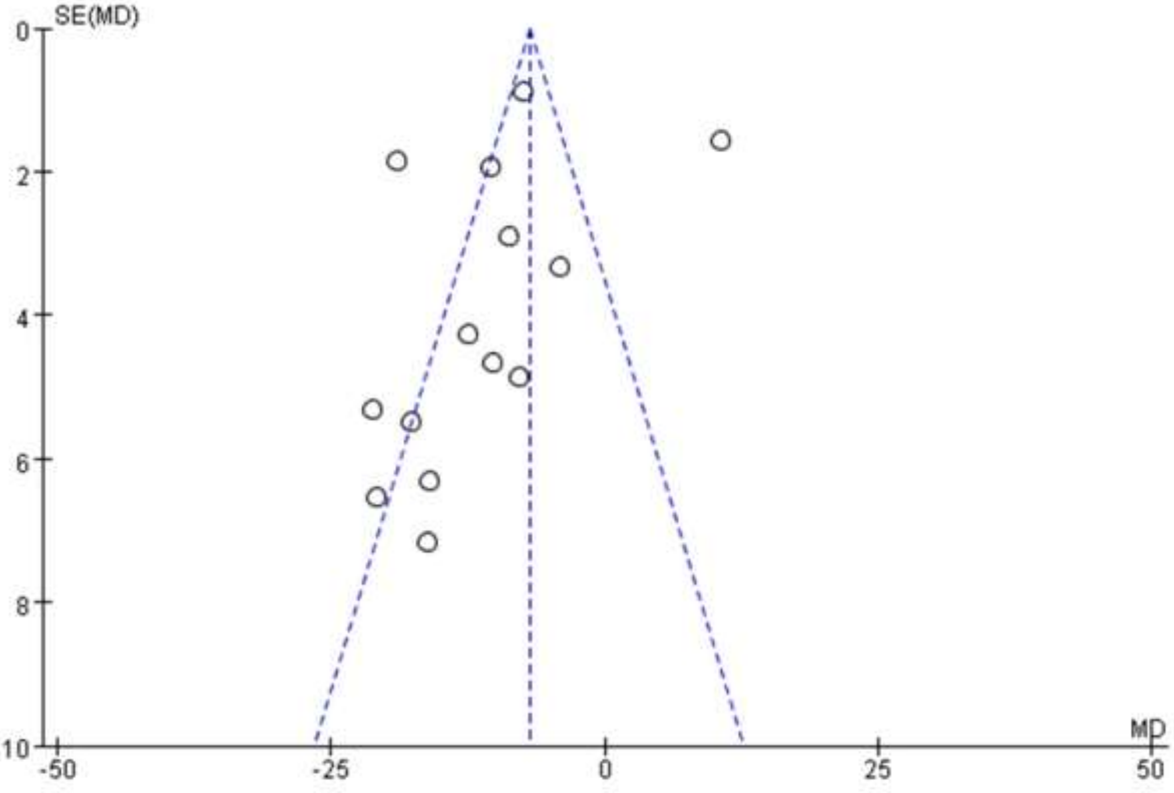

FigueS2 Funnel plot of AST

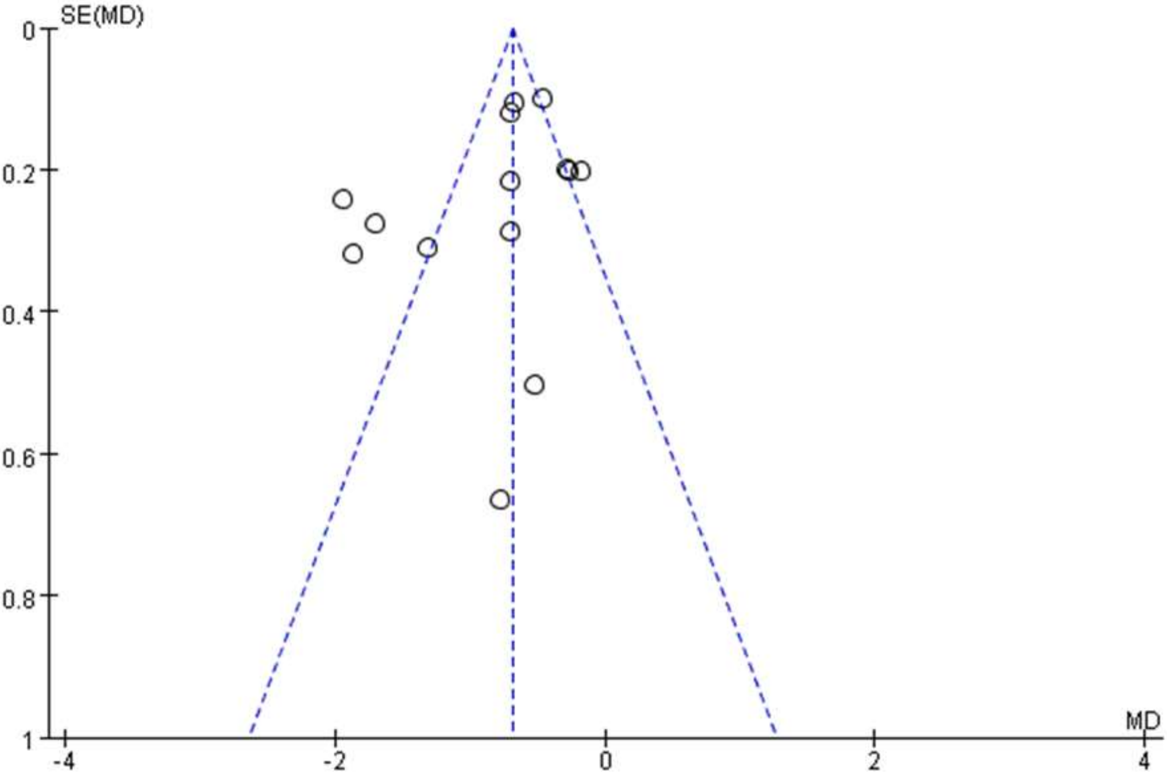

FigueS3 Funnel plot of TC

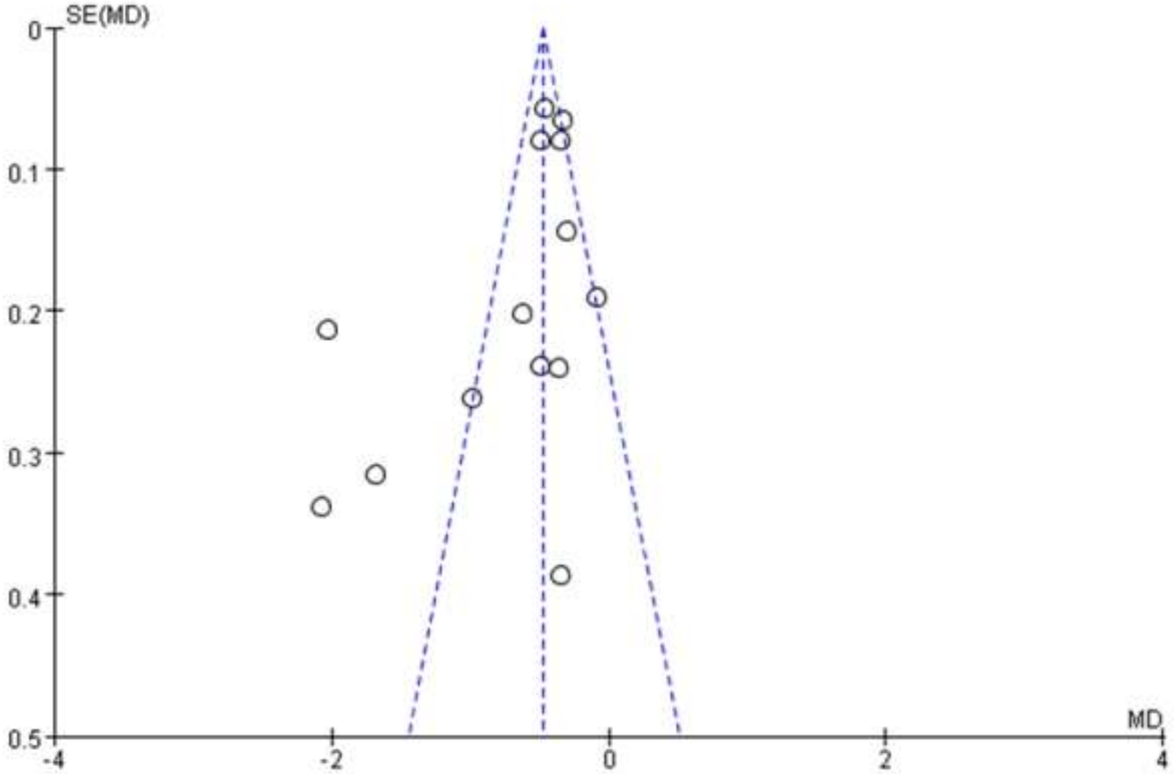

FigueS4 Funnel plot of TG

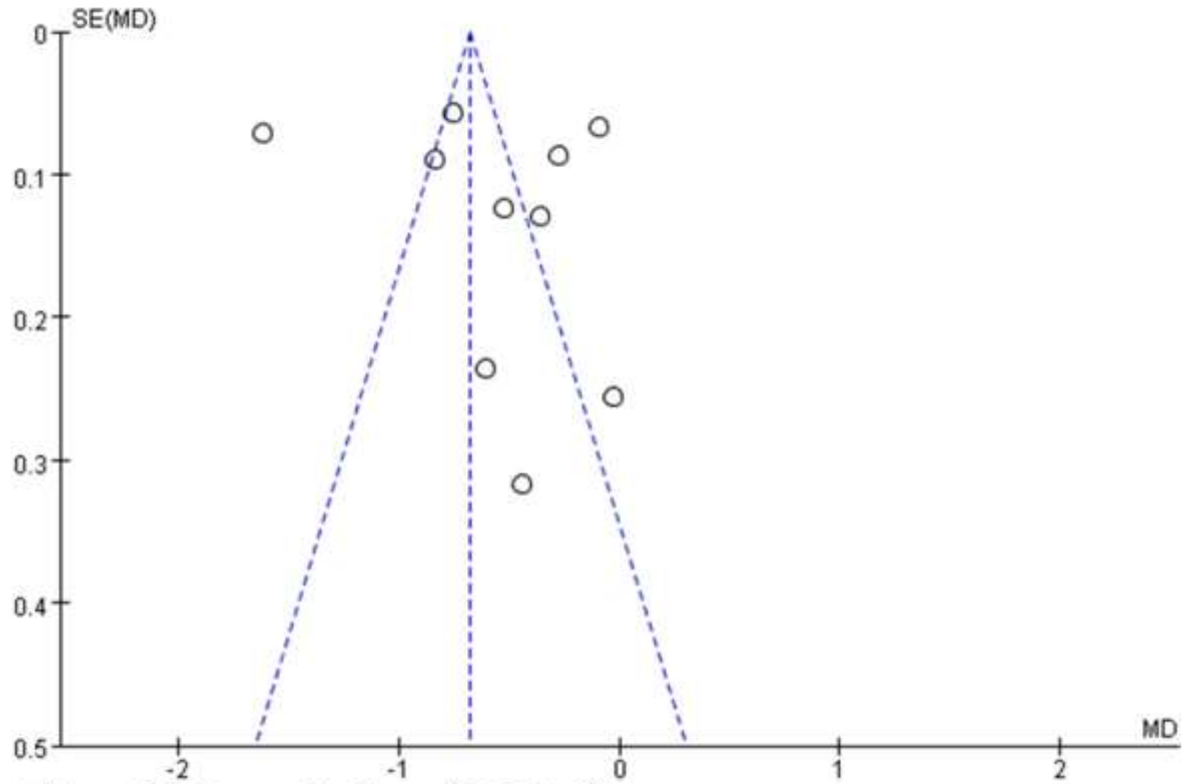

FigueS5 Funnel plot of LDL-C

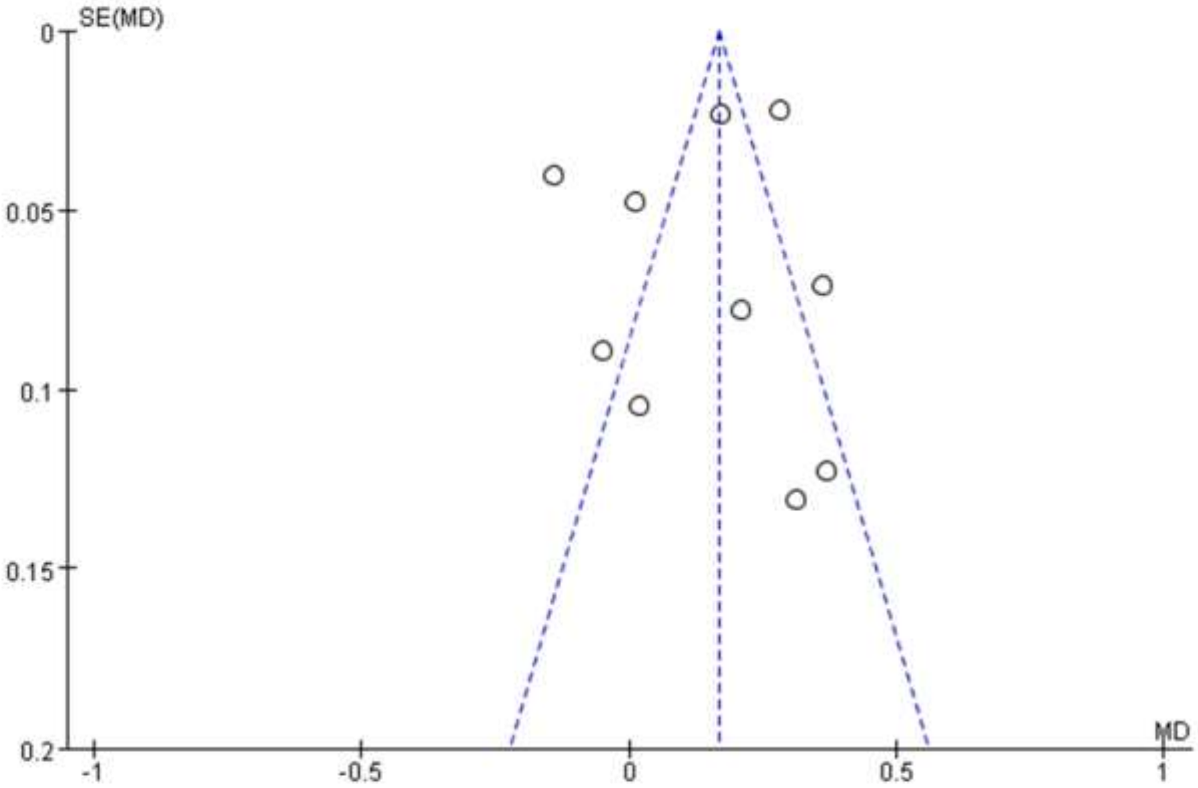

FigueS6 Funnel plot of HDL-C
